# Estimated Cost of Adverse Event Management in Treatment Naïve Non-small Cell Lung Cancer Patients with Common EGFR Mutations Treated with Amivantamab Plus Lazertinib and Osimertinib Plus Chemotherapy

**DOI:** 10.36469/001c.161356

**Published:** 2026-05-18

**Authors:** Alexander Spira, Pratyusha Vadagam, Iris Lin, Courtney Longfield, Robert Musci, Lillian Wang, Denise Zou

**Affiliations:** 1 Virginia Cancer Specialists Research Institute, Fairfax, Virginia; 2 Real-World Value & Evidence Johnson & Johnson, Horsham, Pennsylvania; 3 Real-World Value and Evidence Johnson & Johnson, Horsham, Pennsylvania; 4 Health Economics and Market Access Evidera, Inc., San Francisco, California; 5 Health Economics and Market Access Evidera, Inc., Evidera, Inc., San Francisco, California

**Keywords:** non-small cell lung cancer, EGFR mutations, adverse event management, proactive therapy management, cost analysis, amivantamab plus lazertinib

## Abstract

**Background:**

Recent clinical trials have demonstrated improved efficacy of combination regimens, including amivantamab plus lazertinib and osimertinib plus platinum-based chemotherapy, compared with standard therapy in patients with epidermal growth factor receptor (EGFR)–mutated non-small cell lung cancer (NSCLC). As these regimens enter routine clinical use, understanding the economic implications of treatment-related adverse event (AE) management, including proactive therapy management (PTM), is important.

**Objectives:**

To estimate and compare grade 3 or 4 AE management costs associated with first-line amivantamab (intravenous [IV] or subcutaneous [SC]) plus lazertinib and osimertinib plus platinum-based chemotherapy in patients with EGFR-mutated locally advanced or metastatic NSCLC from a US Commercial and Medicare Advantage payer perspective.

**Methods:**

A descriptive cost modeling analysis used grade 3/4 AE incidence data from pivotal clinical trials of amivantamab (IV and SC) plus lazertinib and osimertinib plus platinum-based chemotherapy. AEs occurring in ≥5% of patients or deemed clinically significant were included. PTM strategies for dermatologic events, venous thromboembolism, and infusion-related reactions were modeled for amivantamab-based regimens using guideline-concordant practices and clinical trial evidence. As a conservative assumption, PTM for osimertinib plus platinum-based chemotherapy was not included. AE management costs were derived from national inpatient and physician fee schedules and inflated to 2025 US dollars. Total per-patient AE management and PTM costs were estimated for each treatment regimen. Scenario analyses evaluated expanded PTM assumptions.

**Results:**

Across both payer perspectives, total AE management costs were estimated to be lower for amivantamab-based regimens than for osimertinib plus platinum-based chemotherapy. Under the Commercial perspective, modeled AE management costs were 2345foramivantamab(SC)pluslazertinib,4321 for amivantamab (IV) plus lazertinib, and 8497forosimertinibplusplatinum−basedchemotherapy.CorrespondingMedicareAdvantageestimateswere1166, 2425,and4185, respectively. Inclusion of PTM costs did not alter the relative cost ranking calculated and findings were consistent across scenario analyses.

**Conclusions:**

Results from this descriptive cross-trial analysis indicated that total AE management costs were consistently estimated to be lower in patients receiving amivantamab (SC or IV) plus lazertinib compared with osimertinib plus platinum-based chemotherapy across both a Commercial and Medicare Advantage payer perspective.

## BACKGROUND

Lung cancer remains one of the most prevalent and devastating malignancies in the United States (US), with non-small cell lung cancer (NSCLC) accounting for approximately 85% of all lung cancer cases.^1^ Current estimates indicate that over 220,000 patients are newly diagnosed with NSCLC annually in the US,^2^ of which 91 152 exhibit locally advanced or metastatic (LA/met) disease,^3^ contributing significantly to the nation’s cancer-related healthcare expenditure.^4^ Among NSCLC cases, mutations in the epidermal growth factor receptor (EGFR) gene, specifically Exon 19 deletions and Exon 21 L858R mutations, are observed in approximately 14% of patients with NSCLC and represent crucial molecular targets for therapy.^5–7^

The current treatment landscape for EGFR-mutated LA/met NSCLC has evolved significantly, with several therapeutic options available for patients with Exon 19 deletions or Exon 21 L858R mutations.^8^ The economic burden of LA/met NSCLC management is substantial, with treatment-related adverse event (AE) management constituting a considerable component of direct treatment costs. The management of treatment-related AEs has emerged as a critical factor influencing treatment decisions for patients with LA/met NSCLC and often necessitate additional diagnostic tests, treatments, and extended hospital stays.^9^

Proactive therapy management (PTM) has emerged as a strategy to mitigate predictable, treatment-related AEs through the anticipatory use of guideline-concordant supportive care.^10^ Unlike reactive approaches that address AEs only after clinical onset, PTM primarily shifts the timing of supportive care earlier in the treatment course, with the goal of reducing event severity, preserving treatment continuity, and limiting downstream healthcare utilization.^9,11^ Importantly, PTM does not introduce additional categories of care, as many supportive interventions are commonly administered under reactive management once AEs occur.^8^

Recent updates to the US National Comprehensive Cancer Network (NCCN) guidelines^8^ have expanded first-line treatment options for EGFR-mutated NSCLC to include amivantamab plus lazertinib and osimertinib plus platinum-based chemotherapy supported by the MARIPOSA/PALOMA-2/PALOMA-3^12–15^ and FLAURA2^16,17^ pivotal trials, respectively (**Table S1**).

Given the evolving treatment landscape and the significant impact of AEs on both patient outcomes and healthcare costs, there is a critical need to understand the economic implications of AE management in EGFR-mutated NSCLC treatment. Therefore, this study aims to estimate the costs associated with grade 3 or 4 AEs for key first-line treatment options, including intravenous (IV) amivantamab plus lazertinib (including recommended PTM), subcutaneous (SC) amivantamab plus lazertinib (including recommended PTM), and osimertinib plus platinum-based chemotherapy from both a US commercial payer and Medicare Advantage payer perspective.

## METHODS

### AE Selection and Analysis Framework

This analysis modeled costs associated with grade 3 and 4 treatment-related AEs in first-line treatment of patients with unresectable, LA/met NSCLC harboring EGFR Exon 19 deletions or Exon 21 L858R mutations. AEs were included in the analysis if they occurred in at least 5% of patients in the corresponding pivotal clinical trials for any one of the select category 1 recommended combination treatment options per the latest NCCN guidelines (ie, for patients receiving erlotinib ± bevacizumab/ramucirumab, gefitinib, afatinib, or dacomitinib for treatment-naïve Exon 19 deletion or Exon 21 L858R+ NSCLC),^8,18–23^ or were deemed clinically significant, such as administration-/infusion-related reactions (ARRs/IRRs), interstitial lung disease, and venous thromboembolism (VTE) (**Table 1**). Clinical significance was defined based on potential to require substantial healthcare resource utilization or to impact treatment delivery in a meaningful way. This analysis was descriptive in nature and was based on AE incidence reported across separate clinical trials. As no head-to-head data were available, results reflect naïve cross-trial comparisons without statistical adjustment.

**Table 1.** Incidence Rates of Grade 3 and 4 AEs Related to Amivantamab (IV) Plus Lazertinib, Amivantamab (SC) Plus Lazertinib, and Osimertinib Plus Platinum-Based Chemotherapy

**Treatment**	**Incidence (%)**		
**Amivantamab (IV) + Lazertinib**	**Amivantamab (SC) + Lazertinib**	**Osimertinib + Platinum-Based Chemotherapy**	
ALT increased	5.013	5.314	1.017
Anemia	3.813	3.5^b^	20.017
AST increased^a^	3.313	3.514	0.417
Dermatitis acneiform^a^	3.728	1.814	0.017
Diarrhea^a^	0.013	1.814	3.017
Dyspnea^a^	1.413	0.0^b^	0.017
Fatigue^a^	0.013	1.8^b^	3.017
Hypermagnesemia^a^	2.613	0.0^b^	0.017
Hypoalbuminemia	5.213	0.014	0.017
Hypokalemia^a^	3.113	1.8^b^	0.017
Hyponatremia	7.413	0.0^b^	0.017
Administration-/infusion-related reaction^a^	2.426	0.0^b^	0.017
Interstitial lung disease^a^	1.413	1.8^b^	0.017
Lymphopenia^a^	0.013	1.8^b^	0.017
Neutropenia	1.413	1.8^b^	13.417
Paronychia^a^	4.828	3.514	1.017
Pneumonia^a^	3.813	0.0^b^	0.017
Rash	12.028	5.314	0.417
Stomatitis^a^	0.013	1.814	0.417
Thrombocytopenia	0.413	0.0^b^	6.917
VTE^a^	3.315	0.0^b^	0.017
Source	Cho 2024,^13^ Girard 2025,^24^ Paz-Ares 2024,^25^ Leighl 2024^15^	Lim 2024,^14^ unpublished observations	Planchard 2023^17^

Abbreviations: AE, adverse event; ALT, alanine aminotransferase; AST, aspartate aminotransferase; IV, intravenous; NCCN, National Comprehensive Cancer Network; SC, subcutaneous; VTE, venous thromboembolism.

^a^Grade 3/4 AEs with incidence <5% in a given treatment arm were included if they met at least one of the following criteria: (1) incidence ≥5% in any comparator regimen from NCCN Category 1-recommended therapies, or (2) deemed clinically significant (eg, administration-/infusion-related reactions, interstitial lung disease, and VTE).

^b^Incidence data for these AEs were sourced from the unpublished observations in the PALOMA-2 trial clinical study report given gaps in published, publicly available sources.

### Proactive Therapy Management

PTM was defined as the preemptive, guideline-concordant use of prophylactic medications and supportive care initiated at or before treatment initiation to prevent or mitigate predictable treatment-related AEs.^11,26,27^ In this analysis, PTM costs and associated impact on AE incidence were specifically assessed for dermatological events, VTE, and ARRs/IRRs in patients receiving amivantamab (SC or IV, respectively) plus lazertinib. Additional trials (eg, COCOON^26^ and SKIPPirr^25^) were conducted to optimize management of patients receiving amivantamab plus lazertinib, and the recommended therapies, dosing regimens, and impact on AE incidence were informed by these studies in alignment with the latest NCCN guidelines.^8^ No guideline-concordant or regimen-specific PTM strategies have been established for osimertinib plus platinum-based chemotherapy.^8,16^ While routine supportive care (eg, antiemetics or corticosteroids) may be administered with chemotherapy,^28^ there is limited publicly available evidence quantifying the impact of PTM on AE incidence or severity in this regimen. In contrast, PTM strategies for amivantamab-based regimens are well-characterized and supported by prospective clinical evidence (eg, COCOON,^26^ SKIPPirr,^25^ and PALOMA^14,15^ trials). Therefore, PTM was modeled only where evidence-based, regimen-specific strategies and associated AE reductions could be robustly parameterized.

Dermatological-related PTM included minocycline administration during the first 12 weeks of treatment, clindamycin 1% lotion from week 13 until treatment discontinuation, and chlorhexidine 4% solution throughout the treatment period in line with the Phase II COCOON trial.^26^

VTE-related PTM utilized oral anticoagulants during the first 4 months of active treatment per the COCOON trial.^26^ Duration of VTE PTM was informed by findings from the MARIPOSA^13^ and PALOMA-3^15^ trials, which identified that most VTE events occurred early in treatment. Patterns of anticoagulant use (50% apixaban and 50% rivaroxaban) were informed by a retrospective claims analysis using the Komodo Research Dataset, a longitudinal US administrative claims database capturing approximately 165 million lives across more than 150 payers with patient characteristics broadly representative of US Census-level demographics (unpublished observations; Komodo KRD Claims Dataset). The analysis covered January 2016 through September 2025 (data cut: August 2025) where 99% of patients receiving first-line amivantamab plus lazertinib with guideline-recommended anticoagulation (N = 75) received apixaban or rivaroxaban.

ARR-/IRR-related management consisted of dexamethasone administration prior to initial amivantamab (SC or IV, respectively) treatment only per the Phase II SKIPPirr trial.^25^

We conducted a scenario analysis, where we modified PTM assumptions to allow up to 10% use of non-direct oral anticoagulant (DOAC), SC enoxaparin despite our real-world claims data showing DOAC use in ≈99% of patients. This conservative scenario is not intended to reflect observed real-world distribution of VTE PTM guideline recommendations,^8^ but rather to test the robustness of our results to plausible deviations in the treatment mix of VTE PTM. Hence, VTE-related PTM under this scenario was assumed as 45% of patients received oral apixaban, 45% received oral rivaroxaban, and 10% received SC-administered enoxaparin. Even with a non-DOAC share of up to 10%, the direction and magnitude of our primary outcomes were unchanged, supporting the stability of our conclusions under alternative, conservative assumptions.

In addition, the expanded ARR/IRR prophylaxis also included dexamethasone (IV) administered alongside the first 2 doses of amivantamab, as well as oral diphenhydramine and acetaminophen administered prior to all amivantamab infusions per the latest amivantamab IV label.^12^

Costs for PTM strategies were calculated based on recommended treatment duration for each PTM intervention and the median treatment duration with amivantamab plus lazertinib observed in the MARIPOSA trial (18.5 months).^13^ Additional detail on the assumptions and calculations to inform the cost of PTM are included in the **Online Supplementary Material** (**Table S2**).

### AE Incidence Rates

Cumulative grade 3 and 4 AE incidence rates identified during the entire patient follow-up were obtained from relevant clinical trials: MARIPOSA trial for amivantamab (IV) plus lazertinib,^12,13^ PALOMA-2 trial (Cohort 6) for amivantamab (SC) plus lazertinib,^14^ and FLAURA2^17^ trial for osimertinib plus platinum-based chemotherapy (**Table 1**). For amivantamab (IV) plus lazertinib, the impact of PTM on dermatological-related AEs was derived from relative reductions observed in the enhanced prophylactic regimen cohort compared with the standard of care cohort in the COCOON trial (44% reduction).^24^ VTE incidence for patients receiving amivantamab (IV) plus lazertinib given the impact of PTM was informed by the Phase III PALOMA-3 trial,^15^ which assessed amivantamab (IV and SC) plus lazertinib in a post-osimertinib and chemotherapy population. In the absence of first-line data, these rates were assumed to be representative of treatment-naïve patients receiving amivantamab (IV) plus lazertinib; however, this introduces uncertainty and should be interpreted with caution. IRR incidence under PTM was derived from the enhanced prophylaxis cohort of the SKIPPirr trial^25^ and was assumed to be applicable to the IV formulation. For amivantamab (SC) plus lazertinib, all grade 3 and 4 AE incidence rates were directly informed by PALOMA-2 (Cohort 6) wherein PTM was mandated per protocol.^14^ While data from the COCOON,^26^ PALOMA-3,^15^ and SKIPPirr^25^ trial represent the most relevant available evidence, differences in patient populations and study designs may limit direct generalizability to the treatment-naïve setting.

### Cost Analysis Methodology

The analysis considered two distinct payer perspectives: US Commercial and Medicare Advantage. For grade 3 and above AEs, management was assumed to occur in the hospital inpatient setting where applicable. However, for certain AEs (eg, dermatologic events and laboratory abnormalities) management was assumed to occur in the outpatient setting only based on clinical expert input indicating that these events, even at Grade ≥3 severity, do not routinely require inpatient admission. While this may not reflect all patient scenarios or reflect the full heterogeneity of real-world management patterns, consistent assumptions were applied across all treatment arms and aligns with a recently published approach.^28^ For the Commercial perspective, inpatient management costs were sourced from the Healthcare Cost and Utilization Project’s National Inpatient Sample,^29^ using corresponding Clinical Classification Software Refined codes. These costs were inflated to 2025 US dollars^30^ given the latest data available are reflective of 2021 US dollars. Medicare Advantage perspective costs were derived from the Centers for Medicare & Medicaid Services (CMS) 2025 Acute Inpatient Prospective Payment System, based on corresponding diagnosis-related group codes (**Table S3**).^31^

Specialist visits costs were obtained from the Practice Management Information Corporation Medical Fees Directory 2025^32^ for the Commercial perspective and the CMS 2025 Physician Fee Schedule^33^ for the Medicare Advantage perspective. AE-related management costs were estimated as expected per-patient costs over the full treatment course by applying cumulative AE incidence rates to corresponding unit costs. Each AE was assumed to incur a single episode of management per patient experiencing the event, consistent with a simplifying assumption of independent, nonrecurrent events.

### Drug Acquisition Costs

For PTM strategies, drug acquisition costs under the Commercial perspective were based on wholesale acquisition cost data from RED BOOK.^34^ For the Medicare Advantage perspective, actual sales price data from the CMS 2025 ASP Pricing File^35^ was used where available. For PTM-related treatments not included in the Medicare Part B list (minocycline, clindamycin 1% lotion, chlorhexidine 4% lotion, apixaban, and rivaroxaban), wholesale acquisition cost data was used as a proxy for acquisition costs under the Medicare Advantage perspective as well. Additional detail on the underlying unit acquisition costs for PTM strategies are included in the supplementary materials (**Table S4**).

## RESULTS

In the amivantamab (SC) plus lazertinib group, the most frequent grade 3 and 4 AEs were rash and increased alanine aminotransferase (ALT; both 5.3%), followed by anemia, paronychia, and increased aspartate aminotransferase (AST; all at 3.5%). In the amivantamab (IV) plus lazertinib group, the most common events were rash (12%), hyponatremia (7.4%), and hypoalbuminemia (5.2%). For osimertinib plus platinum-based chemotherapy, grade 3 and 4 AEs were primarily hematologic, including anemia (20.0%), neutropenia (13.4%), and thrombocytopenia (6.9%; **Table 1**).

**Figure 1.**
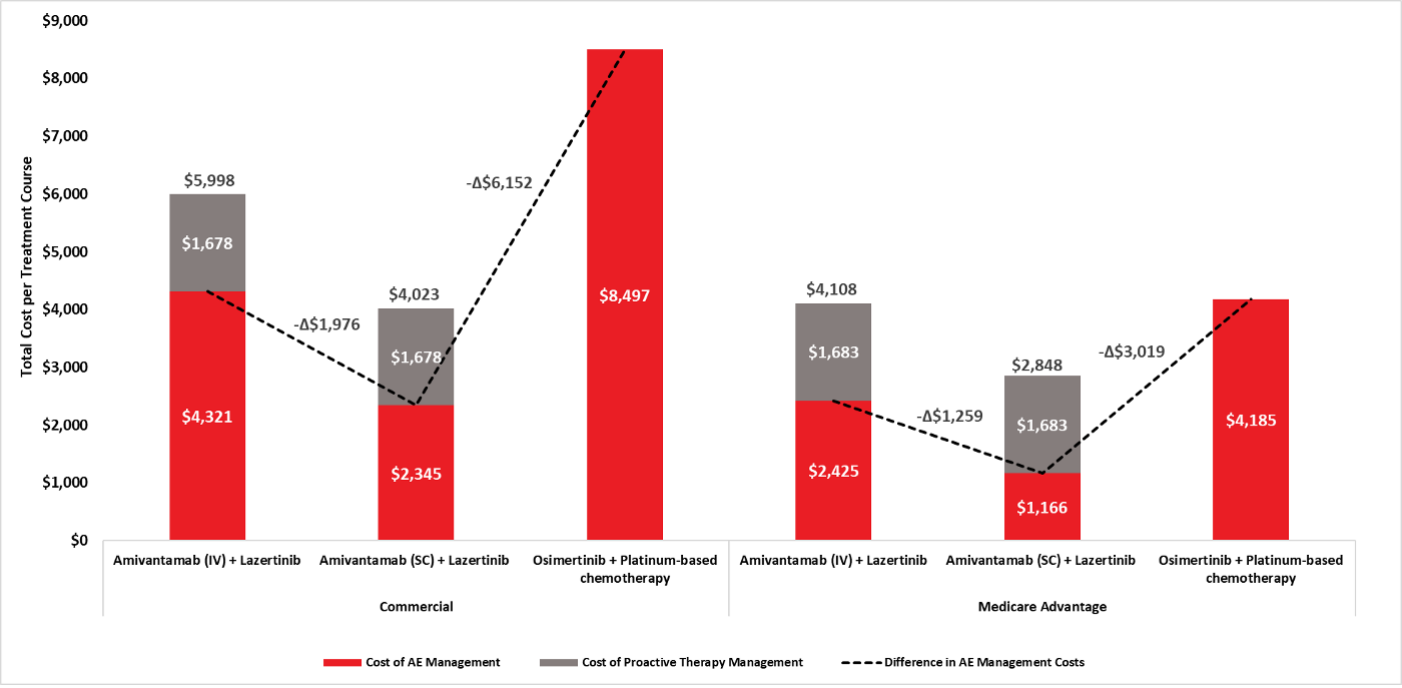
Total Cost of AE Management and Proactive Therapy Management Among Patients Receiving Amivantamab (IV) Plus Lazertinib, Amivantamab (SC) Plus Lazertinib, and Osimertinib Plus Platinum-Based Chemotherapy (Base Case) Abbreviations: AE, adverse event; IV, intravenous; SC, subcutaneous. Dotted lines and delta estimates refer to the difference in total AE management costs for amivantamab (SC) plus lazertinib vs the corresponding treatment arm.

Total AE management cost per patient varied across treatment arm. Under the Commercial perspective, costs were estimated at $2345 for amivantamab (SC) plus lazertinib, $4,321 for amivantamab (IV) plus lazertinib, and $8497 for osimertinib plus platinum-based chemotherapy. Corresponding Medicare Advantage estimates were $1166, $2425, and $4185, respectively (**Table 2; Figure 1**).

**Table 2.** Total AE Management Costs by Treatment Regimen and Perspective

**Treatment-Related AEs**	**Cost ($)**		
**Amivantamab (IV) + Lazertinib**	**Amivantamab (SC) + Lazertinib**	**Osimertinib + Platinum-Based Chemotherapy**	
Commercial perspective			
ALT increased	12	13	2
Anemia	715	660	3763
AST increased	8	8	1
Dermatitis acneiform	9	4	0
Diarrhea	0	181	309
Dyspnea	164	0	0
Fatigue	0	4	7
Hypermagnesemia	6	0	0
Hypoalbuminemia	12	0	0
Hypokalemia	341	193	0
Hyponatremia	813	0	0
Administration-/infusion-related reaction	293	0	0
Interstitial lung disease	399	500	0
Lymphopenia	0	378	0
Neutropenia	302	378	2889
Paronychia	12	8	2
Pneumonia	554	0	0
Rash	29	13	1
Stomatitis	0	4	1
Thrombocytopenia	88	0	1521
VTE	565	0	0
Total cost per patient per treatment course (Commercial perspective)	4321	2345	8497
Medicare Advantage perspective			
ALT increased	6	7	1
Anemia	307	284	1618
AST increased	4	4	1
Dermatitis acneiform	5	2	0
Diarrhea	0	116	198
Dyspnea	81	0	0
Fatigue	0	2	4
Hypermagnesemia	3	0	0
Hypoalbuminemia	7	0	0
Hypokalemia	237	134	0
Hyponatremia	566	0	0
Administration-/infusion-related reaction	201	0	0
Interstitial lung disease	172	216	0
Lymphopenia	0	178	0
Neutropenia	167	209	1598
Paronychia	6	4	1
Pneumonia	316	0	0
Rash	15	7	1
Stomatitis	0	2	1
Thrombocytopenia	44	0	762
VTE	288	0	0
Total cost per patient per treatment course (Medicare Advantage perspective)	**2425**	**1166**	**4185**

Abbreviations: AE, adverse event; ALT, alanine aminotransferase; AST, aspartate aminotransferase; IV, intravenous; SC, subcutaneous; VTE, venous thromboembolism.

Under the Commercial perspective, the principal cost drivers for amivantamab (SC) plus lazertinib were anemia ($660), interstitial lung disease ($500), and neutropenia ($378). For the IV formulation, the largest contributors were hyponatremia ($813), anemia ($715), and VTE ($565). In contrast, anemia ($3763), neutropenia ($2889), and thrombocytopenia ($1521) accounted for the majority of AE costs under osimertinib plus platinum-based chemotherapy. A similar pattern was observed under Medicare Advantage, with anemia ($284), interstitial lung disease ($216), and neutropenia ($209) driving costs for the SC formulation; hyponatremia ($566), anemia ($307), and pneumonia ($316) for the IV formulation; and anemia ($1618), neutropenia ($1598), and thrombocytopenia ($762) for osimertinib plus platinum-based chemotherapy.

PTM costs for amivantamab-based treatment regimens were estimated at $1678 (Commercial) and $1683 (Medicare Advantage) per patient treatment course (**Figure 1; Table S2**). When combined with AE management costs, total per-patient costs under the Commercial perspective were $4023 for amivantamab (SC) plus lazertinib and $5998 for amivantamab (IV) plus lazertinib; corresponding Medicare Advantage totals were $2848 and $4108, respectively. Total costs for osimertinib plus platinum-based chemotherapy were $8497 (Commercial) and $4185 (Medicare Advantage).

Under the scenario analysis considering expanded VTE- and ARR/IRR-related PTM, the cost of PTM in combination with amivantamab (SC or IV) plus lazertinib was estimated to increase to $1975 and $1757 per patient treatment course under Commercial and Medicare Advantage perspectives, respectively (**Figure S1; Table S2**). When combined with AE management costs, total costs for amivantamab (SC) were $4320 (Commercial) and $2923 (Medicare), and $6295 and $4182 for the IV formulation.

Inclusive of PTM, total per-patient treatment course costs under the Commercial perspective ranged from $4023 to $4320 for amivantamab (SC) plus lazertinib and from $5998 to $6295 for amivantamab (IV) plus lazertinib across the base case and scenario analyses, while corresponding costs for osimertinib plus platinum-based chemotherapy remained unchanged at $8497. Under the Medicare Advantage perspective, total costs ranged from $2848 to $2923 for amivantamab (SC) plus lazertinib and from $4108 to $4182 for amivantamab (IV) plus lazertinib, compared with $4185 for osimertinib plus platinum-based chemotherapy.

## DISCUSSION

Recent trials have demonstrated superior efficacy of amivantamab plus lazertinib^13,14^ and osimertinib plus platinum-based chemotherapy^17^ compared with osimertinib monotherapy in EGFR-mutated NSCLC. Given these improved clinical outcomes, understanding the safety profiles and associated management costs is increasingly important.

Results from this descriptive analysis indicated that total AE management costs were consistently estimated to be lower in patients receiving amivantamab (SC or IV) plus lazertinib compared with osimertinib plus platinum-based chemotherapy across both a Commercial and Medicare Advantage payer perspective. Amivantamab (SC) plus lazertinib was associated with the lowest grade 3 or higher AE management costs, reflecting lower rates of VTE and ARRs relative to the IV formulation. Importantly, inclusion of PTM costs did not alter the relative cost ranking across treatment regimens, and findings remained robust in scenario analyses incorporating expanded prophylactic strategies. These findings should be interpreted as exploratory and hypothesis-generating, as comparisons were based on unadjusted data across separate clinical trials with differing patient populations and follow-up durations.

These cost differences reflect underlying safety profiles and the nature of the principal cost drivers observed in this analysis. For osimertinib plus platinum-based chemotherapy, anemia, neutropenia, and thrombocytopenia were the dominant contributors to total AE management costs across both payer perspectives. These hematologic-related AEs frequently require inpatient management, transfusion support, or dose modifications and may recur over multiple cycles, thereby increasing cumulative healthcare resource utilization.^36,37^ In contrast, the primary cost drivers associated with amivantamab-based therapy (such as hyponatremia and VTE for the IV formulation, and anemia and interstitial lung disease for the SC formulation) occurred at lower overall incidence and were generally more predictable and responsive to prophylactic or early supportive care. Evidence from the COCOON,^24^ SKIPPirr,^25^ PALOMA-2,^14^ and PALOMA-3^15^ trials show that PTM strategies can substantially reduce rates of clinically significant rash, VTE, and ARRs/IRRs, ultimately resulting in lower healthcare resource utilization. Given PTM components are also commonly administered under reactive care for AE management, modeled PTM costs may overstate incremental real-world costs, reinforcing the conservative nature of these findings. Finally, prophylactic management may reduce treatment interruptions,^11^ preserve dose intensity,^38^ and decrease healthcare utilization.^39^

A recent analysis by Lopes et al^28^ reached generally consistent conclusions regarding the lower AE burden of amivantamab plus lazertinib; however, several methodological differences warrant consideration. Notably, our analysis conservatively excluded PTM costs for osimertinib plus platinum-based chemotherapy while explicitly incorporating PTM costs for amivantamab-based treatment regimens. In contrast, Lopes et al included PTM costs for osimertinib plus platinum-based chemotherapy (limited to chemotherapy-related pre-medications such as anti-emetics, corticosteroids, and antihistamines) which were estimated at $1660 (Commercial) and $1068 (Medicare) in 2023 US dollars.^28^ Exclusion of these costs in the present analysis may therefore underestimate total supportive care expenditures associated with osimertinib plus platinum-based chemotherapy. Additionally, although Lopes et al incorporated guideline-recommended agents for VTE prophylaxis, their analysis assumed more intensive PTM-related costs than those reflected in current NCCN guidance^8^ and the amivantamab US label,^12^ contributing to higher estimated PTM costs. These differences emphasize the importance of aligning supportive care assumptions with real-world and label-consistent practice.

This study has strengths, including the incorporation of the most up-to-date clinical data and an attempt to quantify both the costs and implications of PTM for amivantamab-based treatment regimens. However, limitations should also be acknowledged. The analysis relied on assumptions regarding the site of care for AE management, with certain conditions (increased AST/ALT, fatigue, hypermagnesemia, hypoalbuminemia, paronychia, proteinuria, rash, and stomatitis) assumed to be managed via a single specialist visit while others were considered as requiring inpatient care. This simplified assignment of site of care (ie, single inpatient vs outpatient visit) may not fully capture real-world variability in AE management, including partial hospitalizations, repeated visits, or escalation of care. These structural assumptions may influence absolute cost estimates, although the relative differences between treatment arms are expected to be directionally consistent. Additionally, the analysis was limited to grade 3 or 4 AEs. This analysis also applied a uniform PTM strategy across both amivantamab (SC) and amivantamab (IV) plus lazertinib treatment regimens. Although core prophylactic strategies are similar, real-world PTM practices may differ between formulations, particularly regarding ARR/IRR-related PTM.

A notable limitation also pertains to the assumption that VTE incidence patterns observed in the post-osimertinib setting for patients receiving amivantamab (IV) plus lazertinib would be representative of first-line treatment setting, which may not fully reflect the clinical reality. PTM was only incorporated for regimens where data on evidence-based, regimen-specific strategies and associated reductions in AE incidence were available. No evidence was identified to support a quantifiable reduction in AE incidence associated with PTM for osimertinib plus platinum-based chemotherapy. In the absence of such data, assuming a clinical benefit from PTM would require unsupported extrapolation and was therefore not incorporated. Additionally, this analysis was inherently descriptive in nature and no indirect treatment comparison or statistical adjustment was conducted, as such approaches would require detailed patient-level data and additional assumptions that were beyond the scope of this analysis. The objective was to provide a transparent, descriptive assessment of AE-related costs. These differences in patient populations and baseline characteristics could potentially confound the observed variations in AE incidence and associated costs which underscores the need for careful interpretation of the comparative findings between cohorts.

Despite these limitations, this analysis provides valuable insights into the economic implications of different treatment approaches and the value of PTM in patients receiving amivantamab plus lazertinib combinations. Given the cross-trial nature of the analysis and the absence of adjustment for baseline differences or exposure duration, the results do not establish definitive comparative cost differences but rather illustrate potential directional trends under a consistent modeling framework. Future research with larger patient populations and more detailed prospective data collection could help address the current limitations and further refine our understanding of these important clinical and economic considerations.

## CONCLUSION

In this descriptive cost modeling analysis, estimated grade 3/4 AE management costs were lower for amivantamab plus lazertinib compared with osimertinib plus platinum-based chemotherapy under the assumptions applied. The SC formulation was associated with the lowest projected AE-related costs. These findings should be interpreted cautiously given the cross-trial design and modeling assumptions, and they primarily highlight the potential economic implications of differing AE profiles and PTM strategies.

### Conflicts of Interest Disclosures

P.V., I.L., and C.L. are employees and stockholders of Johnson & Johnson. R.M., L.W., and D.Z. are employees of Evidera, a company that has provided paid services to Johnson & Johnson, which funded the development and conducting of this analysis.

## Supplementary Material

Online Supplementary Material

## Data Availability

Data sharing is not applicable to this article as no datasets were generated or analyzed during the current study.
