# Supplementary material for: Estimated Cost of Adverse Event Management in Treatment Naïve Non-small Cell Lung Cancer Patients with Common EGFR Mutations Treated with Amivantamab Plus Lazertinib and Osimertinib Plus Chemotherapy
Source: J Health Econ Outcomes Res. 2026 May 18;13(1):174–81. doi: 10.36469/001c.161356 (PMC13189261; doi:10.36469/001c.161356)
Supplement: Online Supplementary Material [file jheor_2026_13_1_161356_345230.pdf]

Journal of Health Economics and Outcomes Research

Online Supplementary Material

Estimated Cost of Adverse Event Management in Treatment Naïve Non-small Cell Lung Cancer Patients with Common EGFR Mutations Treated with Amivantamab Plus Lazertinib and Osimertinib Plus Chemotherapy. *JHEOR*. 2026;13(1):174-181. doi:10.36469/jheor.2026.161356

Table S1: Summary of Relevant Clinical Trials

Table S2: Table S2. Costs of Proactive Therapy Management for Amivantamab (IV and SC) Plus Lazertinib

Table S3: Unit Costs for AE Management

Table S4: Unit Acquisition and Administration Costs for PTM Components

Figure S1: Scenario Analysis for Total Cost of AE Management and Expanded Proactive Therapy Management Among Patients Receiving Amivantamab (IV) Plus Lazertinib, Amivantamab (SC) Plus Lazertinib, and Osimertinib Plus Platinum-Based Chemotherapy

References

This supplementary material has been provided by the authors to give readers additional information about their work.

This is an open-access article distributed under the terms of the Creative Commons Attribution 4.0 International License (CCBY-4.0). View this license's legal deed at http://creativecommons.org/licenses/by/4.0 and legal code at http://creativecommons.org/licenses/by/4.0/legalcode for more information.

Table S1. Summary of Relevant Clinical Trials

Trial Name	Phase	Objective/ITT Population
FLAURA2[1]	Phase III	Evaluated the efficacy of osimertinib monotherapy vs osimertinib combined with platinum-based chemotherapy in treatment-naïve patients with advanced EGFR-mutated NSCLC
MARIPOSA[2]	Phase III	Demonstrated the efficacy of amivantamab in combination with lazertinib; this combination received US FDA approval for the first-line treatment of locally advanced or metastatic NSCLC with EGFR Exon 19del or Exon 21 L858R mutations
PALOMA-2[3]	Phase II	Assessed the efficacy of SC amivantamab combined with lazertinib as a first-line treatment for patients with advanced NSCLC harboring Exon 19del or Exon 21 L858R mutations
PALOMA-3[4]	Phase III	Assessing the noninferiority of pharmacokinetics, efficacy, and safety of subcutaneous vs intravenous amivantamab, both combined with lazertinib, in patients with EGFR-mutated, advanced NSCLC following disease progression on osimertinib and platinum-based chemotherapy.
COCOON[5]	Phase II	Evaluated whether enhanced dermatologic management can reduce incidence of grade greater than or equal to 2 DAEIs when compared with standard-of-care skin management in participants with locally advanced or metastatic stage IIIB/C-IV EGFR-mutated NSCLC treated first-line with amivantamab and lazertinib.
SKIPPirr[6]	Phase II	Assessed the potential of dexamethasone, montelukast and methotrexate administration, prior to amivantamab infusion given through a needle in the vein, to decrease the incidence and/or severity of first-dose infusion related reactions

Abbreviations: DAEI, dermatologic adverse events of interest; EGFR, epidermal growth factor receptor; ITT, intention-to-treat; NSCLC, non-small cell lung cancer; SC, subcutaneous; TKI, tyrosine kinase inhibitor; US FDA, United States Food and Drug Administration.

Table S2. Costs of Proactive Therapy Management for Amivantamab (IV and SC) Plus Lazertinib

Proactive Therapy Management	Commercial Perspective[a], $	Medicare Advantage Perspective[a], $	Duration of Proactive Therapy Management	Summary
Base case				
Dermatologic	451.26	451.26	18.5 months[7]	As minocycline, clindamycin 1% lotion, and chlorhexidine 4% solution are not directly covered by Medicare Advantage (and therefore do not have a corresponding ASP), the model assumes the same monthly cost for individual components of rash prophylaxis under the commercial and Medicare Advantage perspectives.
VTE	1222.07	1222.07	First 4 months of treatment[8]	VTE is applied to patients who remain on treatment with amivantamab + lazertinib for the first 4 months only. As apixaban and rivaroxaban are not directly covered by Medicare Advantage (and therefore do not have a corresponding ASP), the model assumes the same monthly cost for VTE prophylaxis under the commercial and Medicare Advantage perspectives.
IRR	4.50	9.44	Total 5 doses in first month of treatment[6]	IRR is applied to patients who remain on treatment with amivantamab + lazertinib. The one-off cost reflects patients receiving 5 oral administrations of dexamethasone (8 mg) prior to the first administration of amivantamab as part of amivantamab + lazertinib regimen on C1D1 only.
**Total proactive therapy management cost (base case)**	**1678**	**1683**	–	–
Scenario analysis				
Dermatologic	451.26	451.26	18.5 months[7]	Assumed same as base case
VTE	1202.89	1143.37	First 4 months of treatment[8]	VTE is similarly applied to patients who remain on treatment with amivantamab + lazertinib for the first 4 months only based on a weighted average of 45% apixaban, 45% rivaroxaban, and 10% enoxaparin (IV). As apixaban and rivaroxaban are not directly covered by Medicare Advantage (and therefore do not have a corresponding ASP), the model assumes the same monthly cost for VTE prophylaxis under the commercial and Medicare Advantage perspectives.
IRR	320.49	162.56	Dexamethasone (IV and oral): Total 5 doses in first month of treatment;[6] diphenhydramine (oral) and acetaminophen (oral): 18.5 months[7]	IRR is applied to patients who remain on treatment with amivantamab + lazertinib. In addition to the IRR-related proactive therapy management applied in the base case analysis, dexamethasone (IV) administration alongside the first 2 doses of amivantamab and oral diphenhydramine and oral acetaminophen are administered prior to all amivantamab infusions for the full treatment duration.
**Total proactive therapy management cost (scenario)**	**1975**	**1757**	–	–

Abbreviations: ASP, actual sales prices; C1D1, cycle 1, day 1; IRR, infusion-related reaction; IV, intravenous; SC, subcutaneous; VTE, venous thromboembolism.
[a]Total proactive therapy management costs are based on recommended treatment duration for corresponding proactive therapy regimens.

Table S3. Unit Costs for AE Management

Treatment-Related AEs	Commercial Perspective		Medicare Advantage Perspective	
	**CPT/CCSR Code**	**Event Cost, $**	**CPT/DRG Code**	**Event Cost, $**
ALT increased[a]	CPT 99214	240	CPT 99214	125
Anemia	BLD003	18 815	811-812	8091
AST increased[a]	CPT 99214	240	CPT 99214	125
Dermatitis acneiform[a]	CPT 99214	240	CPT 99214	125
Diarrhea	SYM006	10 309	391-392	6607
Dyspnea	SYM013	11 708	204	5788
Fatigue[a]	CPT 99214	240	CPT 99214	125
Hypermagnesemia[a]	CPT 99214	240	CPT 99214	125
Hypoalbuminemia[a]	CPT 99214	240	CPT 99214	125
Hypokalemia	END011	10 984	640-641	7647
Hyponatremia	END011	10 984	640-641	7647
Infusion-related reaction	INJ031	11 999	915-916	8235
Interstitial lung disease	RSP016	28 495	196-198	12 303
Lymphopenia	BLD007	21 560	814-816	10 142
Neutropenia	BLD007	21 560	808-810	11 928
Paronychia[a]	CPT 99214	240	CPT 99214	125
Pneumonia	RSP002	14 577	193-195	8306
Rash[a]	CPT 99214	240	CPT 99214	125
Stomatitis[a]	CPT 99214	240	CPT 99214	125
Thrombocytopenia	BLD006	22 045	813	11 047
VTE	CIR013, CIR033	16 941	175-176, 299-301	8633
Source	HCUP NIS[9]; PMIC Medical Fees Directory[10]		CMS Inpatient Prospective Payment System[11]; CMS Physician Fee Schedule[12]	

Abbreviations: AE, adverse event; ALT, alanine aminotransferase; AST, aspartate aminotransferase; CCSR, Clinical Classifications Software Refined; CMS, Centers for Medicare & Medicaid Services; CPT, current procedural terminology; DRG, diagnosis related group; HCUP NIS, Healthcare Cost and Utilization Project National Inpatient Sample; PMIC, Practice Management Information Corporation; VTE, venous thromboembolism.

[a]Although the analysis only considers incidence and management of grade 3+ AEs, due to the nature of these AEs, management is assumed to be categorized by a single specialist visit rather than in an inpatient setting per clinical input.

Table S4. Unit Acquisition and Administration Costs for PTM Components

**PTM-Related Components**	**Commercial Perspective**			**Medicare Advantage Perspective**	
	**Strength**	**Package Size**	**WAC**	**HCPCS Code Dosage**	**ASP Payment Limit, $**
Dermatologic-related PTM					
Minocycline (oral)	50 mg	100 capsules	22.63	NA[a]	
Clindamycin 1% lotion (topical)	1%	60 mL	40.00	NA[a]	
Chlorhexidine 4% solution (topical)	4%	237 mL	4.55	NA[a]	
VTE PTM					
Apixaban (oral)	5 mg	100 tablets	1010.57	NA[a]	
Rivaroxaban (oral)	20 mg	100 tablets	1993.53	NA[a]	
Enoxaparin (SC)	300 mg/3 mL	3 mL	32.00	10 mg	0.52
ARR-/IRR-related PTM					
Dexamethasone (oral)	4 mg	100 tablets	45.00	0.25 mg	0.06
Dexamethasone (IV)	0.5 mg/5 mL	500 mL	16.17	1 mg	0.09
Diphenhydramine (oral)	50 mg	1000 tablets	13.00	NA[a]	
Acetaminophen (oral)	325 mg	1000 tablets	5.12	NA[a]	
Source	RED BOOK®[13]			CMS 2025 ASP Pricing File[14]	
Administration-related costs, $					
SC injection (CPT 96401)	$278			71	
IV infusion (CPT 96415)	$151			71	
Source	PMIC Medical Fees Directory[10]			CMS Hospital Outpatient PPS[15]	

Abbreviations: ARR, administration-related reaction; ASP, actual sales price; CMS, Centers for Medicare & Medicaid Services; CPT, current procedural terminology; HCPCS, Healthcare Common Procedure Coding System; IRR, infusion-related reaction; IV, intravenous; NA, not applicable; PMIC, Practice Management Information Corporation; PPS, Prospective Payment System; PTM, proactive therapy management; SC, subcutaneous; VTE, venous thromboembolism; WAC, wholesale acquisition price.
aIn the absence of ASP data for treatments not included in the Medicare Part B list, the analysis applies WAC-based pricing was applied under the Medicare Advantage payer perspective as well.

Figure S1. Scenario Analysis for Total Cost of AE Management and Expanded Proactive Therapy Management Among Patients Receiving Amivantamab (IV) Plus Lazertinib, Amivantamab (SC) Plus Lazertinib, and Osimertinib Plus Platinum-Based Chemotherapy

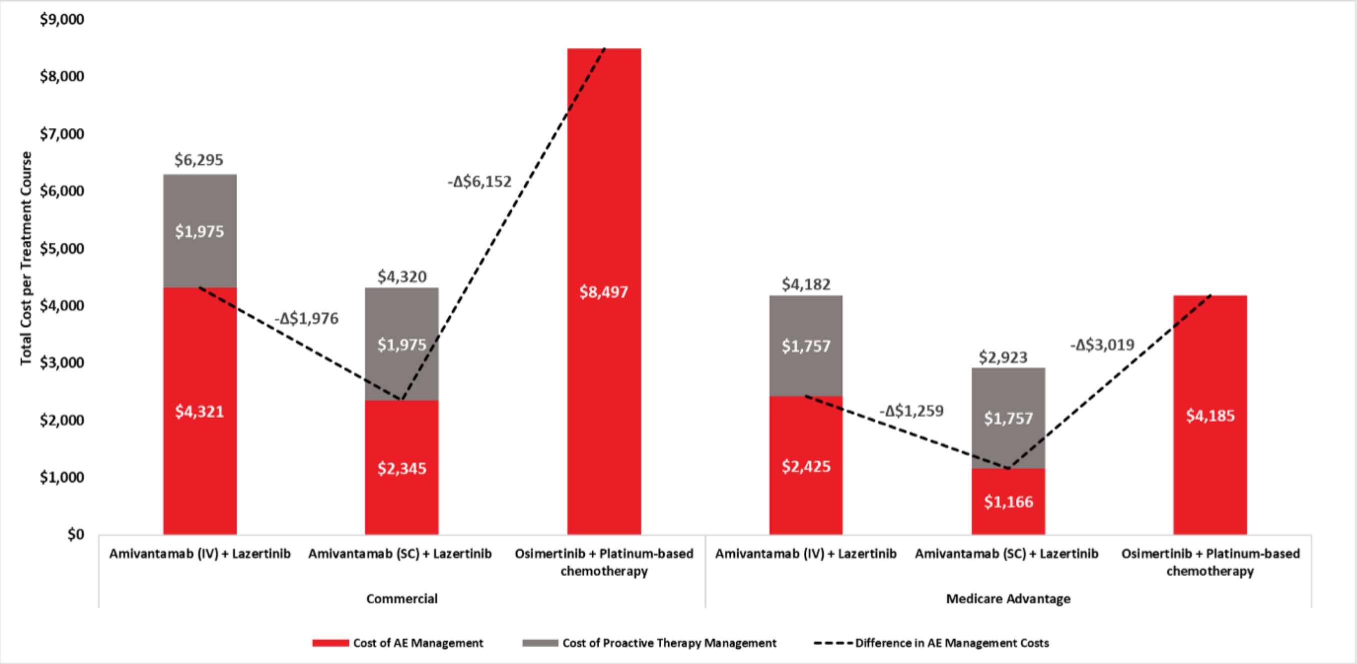

Abbreviations: AE, adverse event; IV, intravenous; SC, subcutaneous.
Dotted lines and delta estimates refer to the difference in total AE management costs for amivantamab (SC) plus lazertinib vs the corresponding treatment arm.

REFERENCES

1. Tagrisso (osimertinib) Prescribing Information. Wilmington, DE; AstraZeneca Pharmaceuticals LP; September 2024. https://www.azpicentral.com/tagrisso/tagrisso.pdf#page=1
2. Rybrevant (amivantamab-vmjw) Prescribing Information. Horsham, PA; Janssen Biotech, Inc.; February 2025. https://www.janssenlabels.com/package-insert/product-monograph/prescribing-information/RYBREVANT-pi.pdf
3. Lim SM, Tan JL, Dias JM, et al. Subcutaneous amivantamab and lazertinib as first-line treatment in patients with EGFR-mutated advanced non-small cell lung cancer (NSCLC): interim results from the phase 2 PALOMA-2 Study. Presented at: American Society of Clinical Oncology (ASCO); May 31-June 4, 2024; Chicago, Ill.
4. Leighl NB, Akamatsu H, Lim SM, et al. Subcutaneous versus intravenous amivantamab, both in combination with lazertinib, in refractory epidermal growth factor receptor-mutated non-small cell lung cancer: primary results from the Phase III PALOMA-3 Study. *J Clin Oncol.* 2024;42(30):3593-3605. doi:10.1200/jco.24.01001
5. ClinicalTrials.gov. Enhanced dermatological care to reduce rash and paronychia in epidermal growth factor receptor (EGRF)-mutated non-small cell lung cancer (NSCLC) treated first-line with amivantamab plus lazertinib (COCOON). Accessed December 2024. https://clinicaltrials.gov/study/NCT06120140?cond=NSCLC&term=COCOON&intr=Amivantamab&rank=5
6. Paz-Ares L, Spira AI, Han JY, et al. Preventing infusion-related reactions with intravenous amivantamab: updated results from SKIPPirr, a phase 2 study. Presented at: European Society for Medical Oncology (ESMO); September 13-17, 2024; Barcelona, Spain.
7. Cho BC, Lu S, Felip E, et al. Amivantamab plus lazertinib in previously untreated EGFR-mutated advanced NSCLC. N Engl J Med. Oct 24 2024;391(16):1486-1498. doi:10.1056/NEJMoa2403614
8. Girard N, Li W, Spira AI, Feldman J, Mak M, Sauder M, et al. Preventing Moderate to Severe Dermatologic Adverse Events in First-line EGFR-mutant Advanced NSCLC Treated with Amivantamab Plus Lazertinib. Early Success of the COCOON Trial. Presented at: European Lung Cancer Congress (ELCC); March 26-March 29, 2025; Paris, France. https://www.jnjmedicalconnect.com/media/attestation/congresses/oncology/2025/elcc/preventing-moderatetosevere-dermatologic-adverse-events-in-firstline-egfrmutant-advanced-nsclc-treat.pdf
9. AHRQ. HCUPnet Inpatient Stays, National statistics by Clinical Classification Software Refined (CCSR), Principal Diagnosis. All Payer 2020 Cost.
10. Practice Management Information Corporation (PMIC). Medical Fees Directory 2025 - eBook. Usual, customary, and reasonable (UCR) Fees. 2025.
11. Centers for Medicare & Medicaid Services. Medicare Acute Inpatient PPS. FY 2025 IPPS Final Rule Home Page: DRG Payment per Stay. 2025.
12. Centers for Medicare & Medicaid Services. Physician Fee Schedule Look-Up Tool. National Payment Amount FY 2025. 2025.
13. Merative Micromedex®. RED BOOK® Online. Accessed February 2025, http://www.micromedexsolutions.com/micromedex2/librarian/
14. Centers for Medicare & Medicaid Services. Medicare Payment Allowance Limits for Medicare Part B Drugs. Q1-Effective January 1, 2025 through March 31, 2025. 2025.
15. Centers for Medicare & Medicaid Services. Hospital Outpatient PPS. Payment Rate by HCPCS Code. Q2 2025. 2025.
